# Training a phage to expand its host range: directed evolution against *Staphylococcus aureus* from diabetic foot infections

**DOI:** 10.1128/aac.00639-26

**Published:** 2026-08-24

**Authors:** Lucile Plumet, Chloé Magnan, Madjid Morsli, Marion Richardoz, Nadia Gaïa, Patrice François, Jean-Philippe Lavigne, Denis Costechareyre, Virginie Molle

**Affiliations:** 1 VBIC, INSERM U1047, Department of Microbiology and Hospital Hygiene, University of Montpellier, CHU Nîmes 36672, Nîmes, France; 2 Genomic Research Laboratory, University Hospitals of Geneva, Service of Infectious Diseases and Faculty of Medicine, Geneva, Switzerland; 3 Greenphage, Cap Alpha, Clapiers, France; The Peter Doherty Institute for Infection and Immunity, Melbourne, Victoria, Australia

**Keywords:** diabetic foot infections, *Staphylococcus aureus*, bacteriophages, phage training, zebrafish model

## Abstract

Diabetic foot infections (DFIs) are a major complication of diabetes frequently involving multidrug-resistant *Staphylococcus aureus*. Their persistence and therapeutic complexity underscore the urgent need for alternatives to conventional antibiotics. Phage therapy offers a promising solution, though its clinical application is often limited by the narrow host range of individual phages. Here, SAVM02, a recently characterized staphylococcal *Kayvirus*, was trained using directed evolution against DFI isolates. After 20 iterative passages on a mixed panel of susceptible and non-susceptible *S. aureus* strains, the trained phage pool (SAVM02-P20) displayed markedly enhanced activity, infecting approximately 77% of tested *S. aureus* isolates and showing partial cross-species activity against coagulase-negative staphylococci. Improved infectivity was linked to increased replication efficiency rather than adsorption. Genomic and phylogenetic analyses further suggested recurrent adaptive changes and preferential host-range expansion within genetically related lineages. *In vivo* testing in a zebrafish embryo model confirmed therapeutic efficacy against a fully susceptible strain but revealed no survival benefit against an initially poorly permissive isolate, highlighting the gap between *in vitro* adaptation and *in vivo* efficacy. These findings demonstrate both the potential and the limitations of phage training to expand therapeutic coverage and emphasize the need to integrate evolutionary approaches with physiologically relevant models to optimize phage therapy for chronic infections, such as DFIs.

## INTRODUCTION

Diabetic foot infections (DFIs) represent one of the most severe and costly complications of diabetes mellitus, posing a major public health burden due to their high prevalence, chronic nature, and clinical complexity. These infections frequently lead to extended hospital stays, repeated courses of antibiotics, and, in the most severe cases, limb amputation (1). Among the pathogens commonly isolated from DFIs, *Staphylococcus aureus* remains predominant owing to its ability to colonize chronic wounds, form biofilms, and evade host immune defenses. Of particular concern is the prevalence of antibiotic-resistant strains, notably methicillin-resistant *S. aureus* (MRSA), highlighting the urgent need for innovative, effective, and sustainable therapeutic strategies (2–4).

Phage therapy first introduced in the early 20th century has regained scientific interest as a potential alternative to antibiotics in the era of escalating antimicrobial resistance. Virulent bacteriophages, viruses that specifically infect and lyse bacteria, offer several advantages, including high specificity for bacterial targets, self-replicating properties at the infection site, a favorable safety profile, and antibiofilm activity (5, 6). Recent studies have demonstrated the efficacy of phages against *S. aureus* in *in vitro* and *in vivo* models of DFI, as well as in compassionate clinical use, reinforcing their therapeutic potential (7, 8). However, a major limitation of phage therapy lies in the typically narrow host range of individual phages, which can restrict their effectiveness against genetically diverse or non-susceptible clinical isolates. To overcome this, personalized strategies, such as directed evolution, have been explored to broaden phage host range and improve infectivity to clinically relevant bacterial strains (9, 10). Among these, the Appelmans protocol derived from the liquid phage titration method developed by René Appelmans in the 1920s and later adapted for phage training by Burrowes et al. (11) has proven effective in selecting phage variants with improved infectivity and expanded host spectra through iterative co-culture cycles with bacterial hosts. Despite encouraging *in vitro* results, few studies have evaluated trained phages against diverse collection of clinical *S. aureus* isolates, and even fewer have investigated their performance in *in vivo* models (12, 13).

In this study, we adapted this protocol for a single phage, implementing directed evolution (phage training) to broaden the activity of SAVM02, a recently characterized staphylococcal *Kayvirus*, against clinical DFI isolates. Beyond assessing the expanded host range, we combined phenotypic assays, genomic characterization of trained phages, phylogenetic analyses of bacterial hosts, and a zebrafish embryo infection model to investigate the mechanisms and therapeutic relevance of phage training. Our findings provide new insights into the potential and limitations of phage training strategies for tailoring phage therapy to chronic, difficult-to-treat infections, such as DFIs.

## MATERIALS AND METHODS

### Bacterial strains and phage

Clinical isolates were collected between 2018 and 2024 from patients with DFI or diabetic foot osteomyelitis (DFOM) who were mainly hospitalized at the Gard-Occitanie Diabetic Foot Clinic (University Hospital of Nîmes, France), as well as at other hospitals in France and one in Switzerland. The collection included 31 *S. aureus* strains and 28 coagulase-negative staphylococci (CoNS). Staphylococcal isolates were cultured for 24 h at 37°C in tryptic soy broth (TSB) or on tryptic soy agar (TSA). The *Kayvirus* SAVM02 phage previously isolated and characterized by Plumet et al. (8) was propagated on the DFI isolate *S. aureus* NSA1385 as described.

### Whole-genome sequencing and phylogenetic analysis of clinical isolates

Genomic DNA was extracted from the 59 staphylococcal isolates using the DNeasy Ultra Clean Kit (Qiagen, Courtaboeuf, France) following the manufacturer’s protocol and eluted in 50 µL. DNA concentration was measured with a Qubit fluorometer using Qubit dsDNA High-Sensitivity Assay Kit (Life Technologies, Villebon-sur-Yvette, France). Whole-genome sequencing (WGS) libraries were prepared using the Illumina DNA Prep Kit (Illumina, San Diego, USA) and sequenced on an Illumina MiSeq platform with a 39-h run, producing 2 × 250 bp paired-end reads, as previously described (14). Raw sequencing reads were quality-controlled with FastQC (v0.12.1) and assembled *de novo* using Unicycler (v0.4.8). Species identification was performed by whole-genome alignment against the National Center for Biotechnology Information (NCBI) GenBank database using BLASTn confirmed via the Type Strain Genome Server (TYGS) platform (https://tygs.dsmz.de/) and by computing the Average Nucleotide Identity (ANI) with OAT (v0.93.10). Methicillin resistance was identified using ResFinder (v0.7.2). Whole-genome alignment was performed using MAFFT (v7), and a phylogenetic tree was constructed from the aligned sequences in SeaView (v5.0.5) using the Neighbor-Joining method with Kimura-2 parameters and 1,000 bootstrap replicates. The resulting tree was visualized with iTOL (https://itol.embl.de/). Anti-phage defense systems were identified in all clinical isolates using DefenseFinder (15–18).

### SAVM02 phage training

To broaden the host range of SAVM02, we applied a directed evolution protocol by Burrowes et al. (11), adapted here for a single phage rather than multiple phages. Briefly, in a 96-well plate, 20 µL of serial 10-fold phage dilutions (ranging from 10^0^ to 10^−9^) was added to columns 1 to 10, with each column corresponding to a specific dilution. Column 11 received 20 µL of SM buffer as a phage-free growth control. Overnight cultures of eight *S*. *aureus* DFI isolates (four susceptible to SAVM02: NSA1385, SAC1, C01P007Pied, C01P013Pied; and four classified as non-susceptible to SAVM02: NSA739, C01P007Nez, C1BP006Pied, SAC2) were adjusted to an optical density at 600 nm (OD_600_) of 0.2. Then, 180 µL of each bacterial suspension was dispensed into rows 1 to 11, with one strain per row. Column 12 was filled with 180 µL of TSB as a negative control. Plates were incubated at 37°C and 108 rpm for 17 h in a Tecan Spark plate reader (Grödig, Austria). Wells showing complete or partial lysis compared to the growth control, as well as the first well with complete turbidity, were harvested and pooled. The resulting lysate was centrifuged at 4,000 rpm for 10 min at 4°C and filtered through a 0.22 µm syringe filter to obtain the passage 1 (P1) phage training pool. This pool was subsequently used to inoculate a new 96-well plate containing fresh cultures of the same bacterial strains for passage 2 (P2). This iterative process was repeated up to passage 20 (P20). All phage training pools were stored at 4°C, and pools from passages P5, P10, P15, and P20 were selected for further experiments.

### Lytic activity and host range of trained phages

Phage activity was assessed by spot test assay. Briefly, 5 µL of serial 10-fold dilutions of phages was spotted onto TSB-soft agar overlays (TSB containing 0.6% agar) inoculated with overnight bacterial cultures. After 18 h at 37°C, plaque-forming units (PFUs) were enumerated. The efficiency of plating (EOP) was calculated by dividing the phage titer obtained on the test strain by the titer on the reference strain used for phage propagation. Strains were considered phage-susceptible when the EOP was >0.001 (19, 20).

To further assess phage lytic activity, growth inhibition assays were performed in liquid culture at a multiplicity of infection (MOI) of 0.1. Bacterial growth was monitored over time by measuring OD_600_ in a Tecan Spark plate reader (Grödig, Austria). The liquid assay score (LAS) was calculated as described by Xie et al. (21) by comparing the area under the bacterial growth curve in presence or absence of phage. Strains were considered phage-susceptible when the LAS was >10.

### Adsorption rate

The phage adsorption rate was evaluated through an adsorption assay. *S. aureus* DFI isolates were cultured in 10 mL TSB at 37°C with shaking at 180 rpm until reaching an OD_600_ of 0.4 (approximately 10^8^ CFU/mL). Phages were added to the bacterial culture at an MOI of 0.01 (10^6^ CFU/mL) and incubated at 37°C without shaking. Samples were collected every 10 min over a 60 min period and centrifuged at 12,000 rpm for 3 min to pellet the adsorbed phages. The supernatants were then serially diluted and titrated to quantify the remaining non-adsorbed phages.

### Phage replication dynamics assay

The kinetics of phage amplification were evaluated by monitoring phage titers over time. *S. aureus* cultures were grown to an OD_600_ of 0.4 and infected with phages at an MOI of 0.01. The mixture was incubated at 37°C for 10–15 min without shaking to facilitate phage adsorption. Cultures were then centrifuged at 4,000 rpm for 10 min, and the pellet was resuspended in 10 mL of TSB and incubated at 37°C with shaking at 180 rpm. Samples were collected every 30 min for 4 h for phage titration.

### Isolation and sequencing of trained phage isolates

After 20 rounds of phage training, 100 µL of the P20 phage pool was mixed with 100 µL of an overnight culture of each of the four initially non-susceptible *S. aureus* strains included in the training protocol, along with 4 mL of medium for the double agar overlay technique. From each strain, three individual plaques were isolated and purified through three successive rounds of plaque picking. The resulting 12 phage isolates were then amplified on their respective bacterial hosts or on the SAVM02 production strain, and their infectivity was assessed using EOP assays.

For WGS, DNA was extracted from 2 mL of phage suspensions for which sufficient DNA of adequate quality was obtained, following overnight treatment with Benzonase (1 mU/µL). Benzonase was subsequently heat-inactivated at 95°C for 20 min. Samples were then treated with Proteinase K for 5 h, followed by heat inactivation and DNA purification using the DNeasy Kit (Qiagen) according to the manufacturer instructions. DNA quality was assessed prior to library preparation. Libraries were prepared using the TruSeq kit (Illumina) with barcoding following the manufacturer recommendations and sequenced on the Illumina HiSeq platform (Illumina, San Diego, CA, USA), generating 100 bp paired-end reads. Read quality was evaluated using Trimmomatic v0.36 with the following parameters for paired-end reads: SLIDINGWINDOW:10:30 (sliding window size and mean quality threshold) and MINLEN:80 (minimum read length). Sequence variants were identified using Snippy v4.6.0, with the SAVM02 wild-type genome as the reference.

### Zebrafish infection model

Zebrafish (AB wild-type) were reared and staged as previously described (22, 23), and experiments were conducted in compliance with the European Union Directive 2010/63/EU on the protection of animals used for scientific purposes. Embryos obtained from natural spawning of adult zebrafish and used before reaching the free-feeding stage (120 h post-fertilization, hpf) are not considered animal experiments. *S. aureus* aliquots were prepared as previously described and stored at −80°C (24). On the day of use, bacterial aliquots were resuspended in PBS with 0.1% phenol red and 60 µM fluorescent dye dextran. Zebrafish embryos were injected at 30 hpf with 1 nL of this suspension (10^4^ CFU/embryo) into the hindbrain ventricle. Two hours later, phage suspensions (10^9^ PFU/mL in 0.1% phenol red and 60 µM dextran) were injected at the same site. To determine the inoculum dose, 10 euthanized embryos were mechanically homogenized in 200 µL of 1X Triton. The resulting homogenate was serially diluted and plated on TSA for CFU enumeration. For PFU quantification, each dilution was mixed with an overnight culture of the SAVM02 propagation strain and TSB-soft agar, then poured as an overlay onto TSA plates using the double-layer agar method. Embryo survival was monitored up to 72 h post-treatment (hpt); embryo mortality was defined as the absence of heartbeat and blood circulation.

### Statistical analysis

Statistical analyses were performed using GraphPad Prism version 10.6.0. A *P*-value < 0.05 was considered statistically significant. Specific statistical tests used for each experiment are described in the corresponding figure legends.

## RESULTS

### Phage training enhances the lytic activity of SAVM02 against previously non-susceptible *S. aureus* isolates

**Fig 1:**
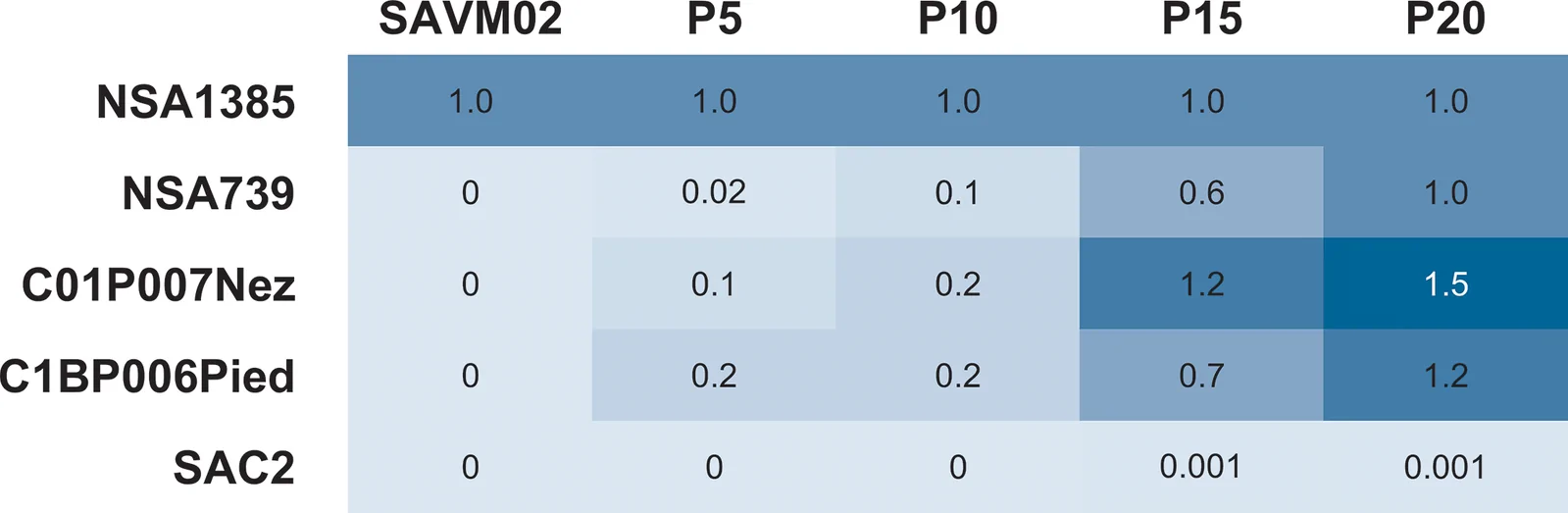
Activity of ancestral SAVM02 and trained phage pools against *S. aureus* isolates based on efficiency of plating (EOP). Strains were considered phage-susceptible when EOP was greater than 0.001.

To improve the infectivity profile of SAVM02, which initially exhibited limited activity against several clinical *S. aureus* isolates from DFIs and DFOMs, we applied phage training using a directed evolution protocol adapted for a single phage. Eight *S*. *aureus* strains were selected as the training panel, comprising four strains susceptible to SAVM02 (NSA1385, SAC1, C01P007Pied, C01P013Pied) and four strains non-susceptible to SAVM02 (NSA739, C01P007Nez, C1BP006Pied, SAC2). The panel was designed to include both permissive and non-permissive isolates belonging to related sequence types (ST), notably ST5 and ST8, with the aim of facilitating progressive phage adaptation across genetically related host backgrounds. The phage was co-cultured with this panel over 20 sequential passages. At each passage, lysates from wells exhibiting lysis were pooled and used to infect fresh bacterial cultures for the next round. At successive stages (P5, P10, P15, and P20), EOP values were determined using spot tests to monitor changes in infectivity relative to the ancestral phage SAVM02 across the propagation strain and the four initially non-susceptible isolates (Fig. 1). While ancestral SAVM02 showed no activity against NSA739, C01P007Nez, and C1BP006Pied, EOP values progressively increased during training (P5, P10, P15), reaching levels at P20 comparable to or exceeding those observed on the propagation strain NSA1385 (EOP ≥ 1). By contrast, SAC2 remained non-permissive throughout the training process.

**Fig 2:**
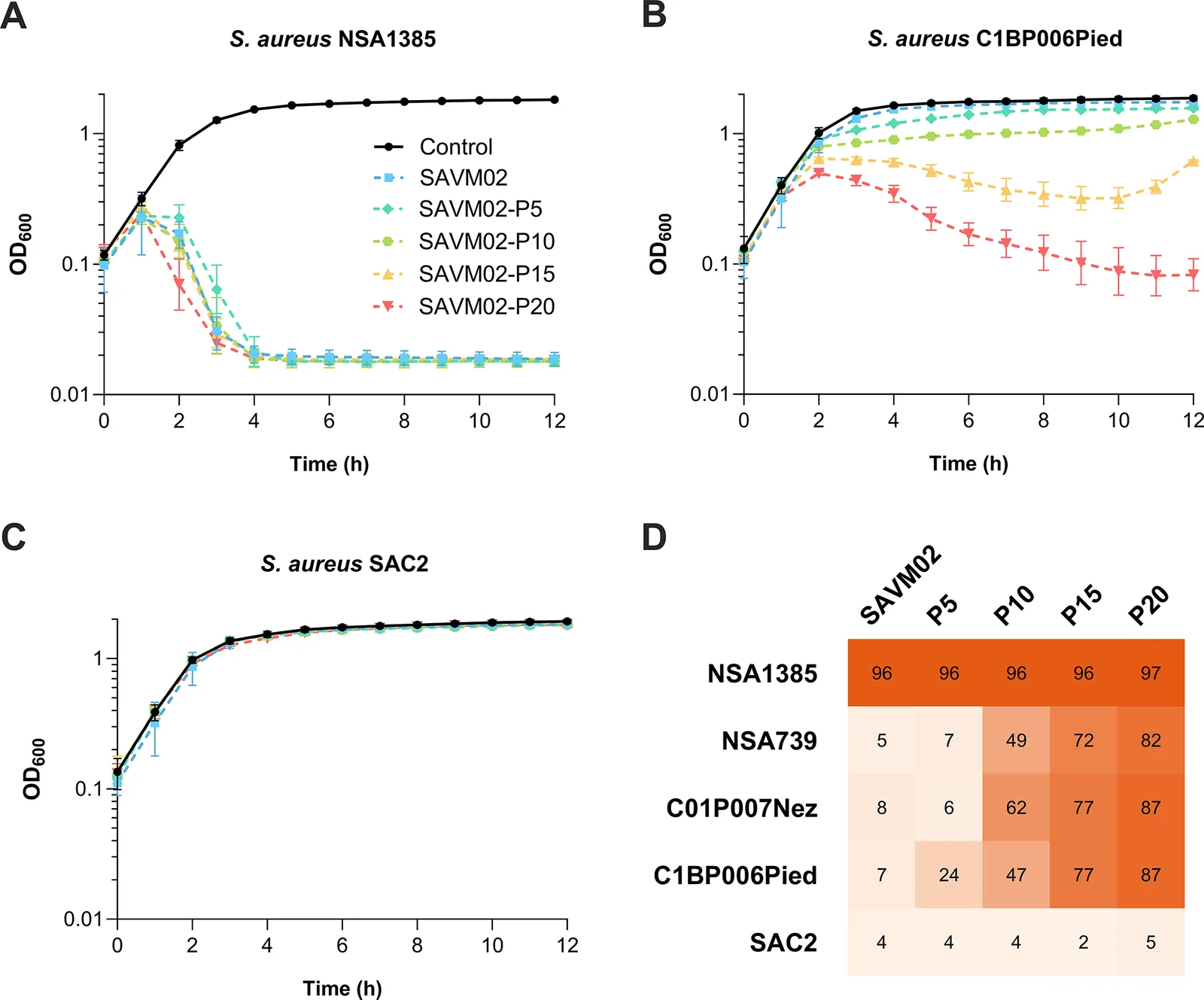
Growth inhibition of clinical *S. aureus* isolates by ancestral SAVM02 and trained phage pools. Bacterial growth of NSA1385 (**A**), C1BP006Pied (**B**), and SAC2 (**C**) was monitored over 12 h in the presence of the ancestral SAVM02 phage, trained phage pools (P5, P10, P15, P20), or without phage (control). At each time point, the data represent the mean OD_600_ readings ± SD of three independent experiments. (**D**) Liquid assay score (LAS) calculated from the area under the bacterial growth curve in the presence or absence of phage. Strains were considered phage-susceptible when LAS was greater than 10. Growth curves for NSA739 and C01P007Nez are shown in Fig. S1.

To evaluate the functional impact of phage training, we performed kinetic growth inhibition assays in liquid culture and calculated LAS values. Each condition was tested in triplicate using an MOI of 0.1, and bacterial growth was monitored over 12 h by measuring OD_600_. As expected for a fully susceptible strain, the ancestral phage SAVM02 completely inhibited the growth of NSA1385 (Fig. 2A), whereas no inhibition was observed for the non-susceptible isolates. After training, however, bacterial strains C1BP006Pied (Fig. 2B), NSA739, and C01P007Nez (Fig. S1) exhibited progressive inhibition from SAVM02-P5 to SAVM02-P20. This observation was also reflected quantitatively, as LAS, calculated as the reduction of growth relative to the untreated control, increased progressively at each passage (Fig. 2D). For instance, LAS increased from 5 to 8 initially to values above 80 by P20, reflecting strong lytic activity. By contrast, SAC2 remained entirely non-permissive to all phage versions, with growth curves indistinguishable from the uninfected control (Fig. 2C) and LAS consistently <10.

Collectively, the results of spot tests and kinetic growth inhibition assays suggest that phage training substantially enhanced the lytic activity and infectivity profile of SAVM02 against three previously non-susceptible clinical isolates, C1BP006Pied, NSA739, and C01P007Nez, belonging to the ST5 and ST8 lineages, whereas the ST15 isolate SAC2 remained refractory throughout the protocol.

### Phage training expands host range among DFI-associated staphylococcal isolates

**Fig 3:**
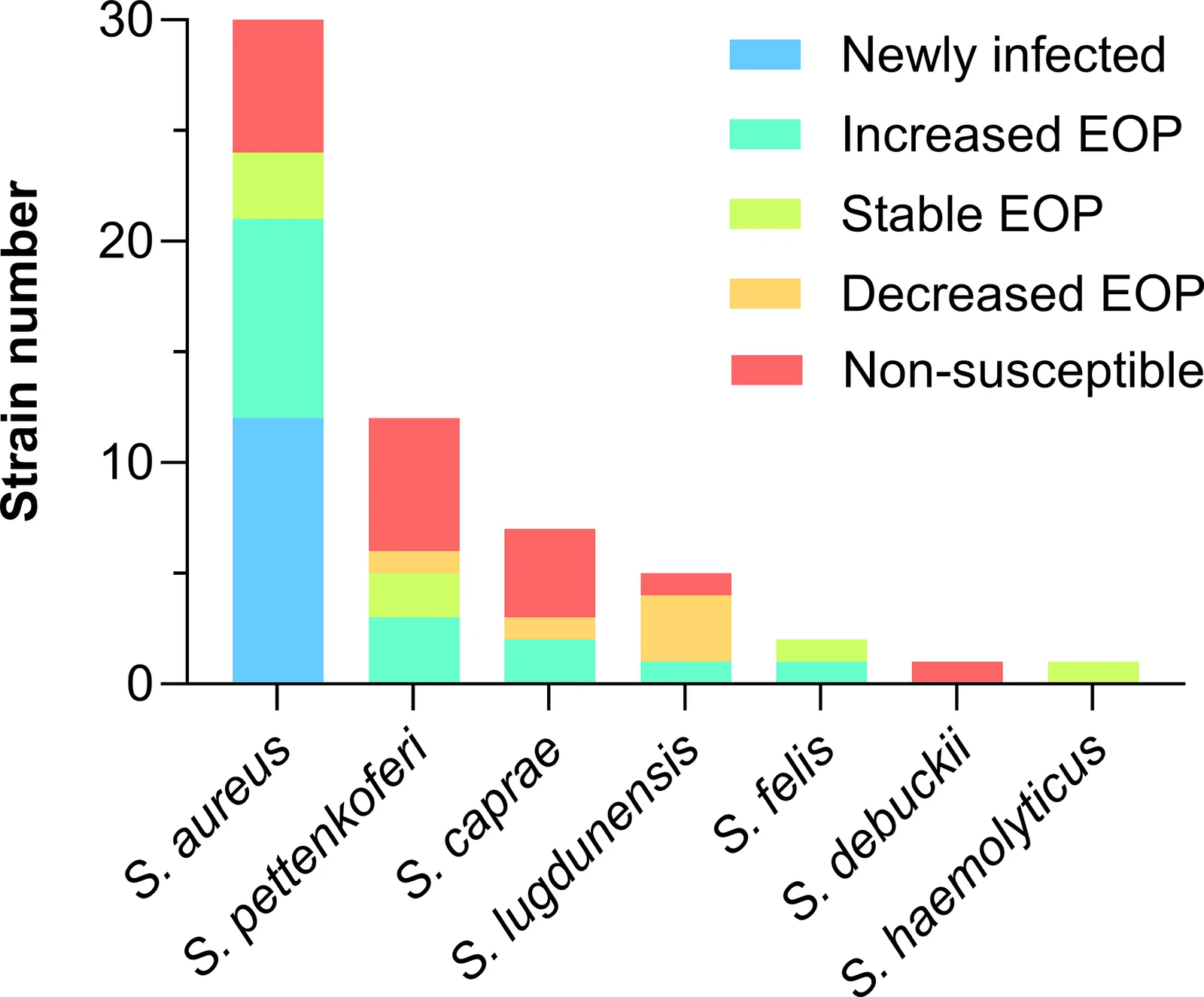
Infectivity profiles based on EOP results for ancestral and trained SAVM02 phage across 59 clinical staphylococcal isolates. Bar chart shows the distribution of infectivity categories based on EOP assays. Categories were defined using an EOP threshold of 0.1 between SAVM02 and SAVM02-P20 as follows: *newly infected* refers to strains initially non-susceptible to the ancestral phage that became susceptible after phage training; *increased EOP* includes strains already susceptible to the ancestral phage that showed stronger lytic activity with the trained phage; *stable EOP* describes strains that remained susceptible, with comparable infectivity for both phage versions; *decreased EOP* corresponds to strains showing reduced infectivity with the trained phage compared to the ancestral one; and *non-susceptible* refers to strains that were not infected by either phage.

To determine whether the improvements acquired during phage training translated into broader host range, we assessed the activity of both ancestral and trained SAVM02 against 59 clinical staphylococcal isolates relevant to DFIs and DFOMs. Isolates were categorized based on EOP outcomes as newly infected, increased EOP, stable EOP, decreased EOP, or non-susceptible using an EOP threshold of 0.1 between SAVM02 and SAVM02-P20 (Fig. 3).

Among *S. aureus* isolates, SAVM02-P20 exhibited a markedly expanded host range. Twelve strains previously non-susceptible to SAVM02 became permissive to infection with the trained phage pool, showing newly detectable lytic activity and EOP values exceeding the 0.001 threshold. Additionally, nine strains that were already sensitive to SAVM02 displayed increased susceptibility, while three remained stably sensitive, with comparable EOPs for both phage versions. Seven isolates remained non-permissive to either phage. Overall, 24 of the 31 *S. aureus* clinical isolates were susceptible to SAVM02-P20 (77.4%) compared with 12 of 31 for the ancestral phage (38.7%), corresponding to an approximate 39% increase in host range. Importantly, no loss of activity was observed on previously *S. aureus-*susceptible strains, suggesting that phage adaptation did not compromise therapeutic potential. A Chi-square test with Yates’ correction confirmed a significant increase in susceptibility following phage training (*χ*² = 8.0, *P* = 0.0046).

Although this study primarily focused on *S. aureus*, the panel also included 28 CoNS isolated from DFIs and DFOMs. In total, 16 of the 28 CoNS isolates (57%) were susceptible to both SAVM02 and SAVM02-P20, with no significant difference in overall infectivity between the two phages (*χ*² = 0, *P* = 1). Among susceptible isolates, several showed moderate changes in lytic activity following training. Improved activity was observed for three *S*. *pettenkoferi*, two *S*. *caprae*, one *S*. *lugdunensis*, and one *S. felis* isolate, whereas reduced EOP values were observed for a small number of CoNS isolates, although these strains remained susceptible to SAVM02-P20. No previously resistant CoNS isolate became susceptible to the trained phage, indicating the absence of *de novo* host-range expansion across species. Overall, SAVM02-P20 exhibited limited but detectable cross-species effects.

When combining species, SAVM02-P20 infected 40 of the 59 tested isolates (67.8%) compared with 28/59 (47.5%) for SAVM02, confirming a significant global increase in infectivity (*χ*² = 4.2, *P* = 0.04). These findings indicate that host-range expansion was primarily restricted to *S. aureus*, while cross-species infectivity toward CoNS remained limited.

### Association of SAVM02 and SAVM02-P20 susceptibility with phylogenetic background and defense system profiles in staphylococcal isolates

**Fig 4:**
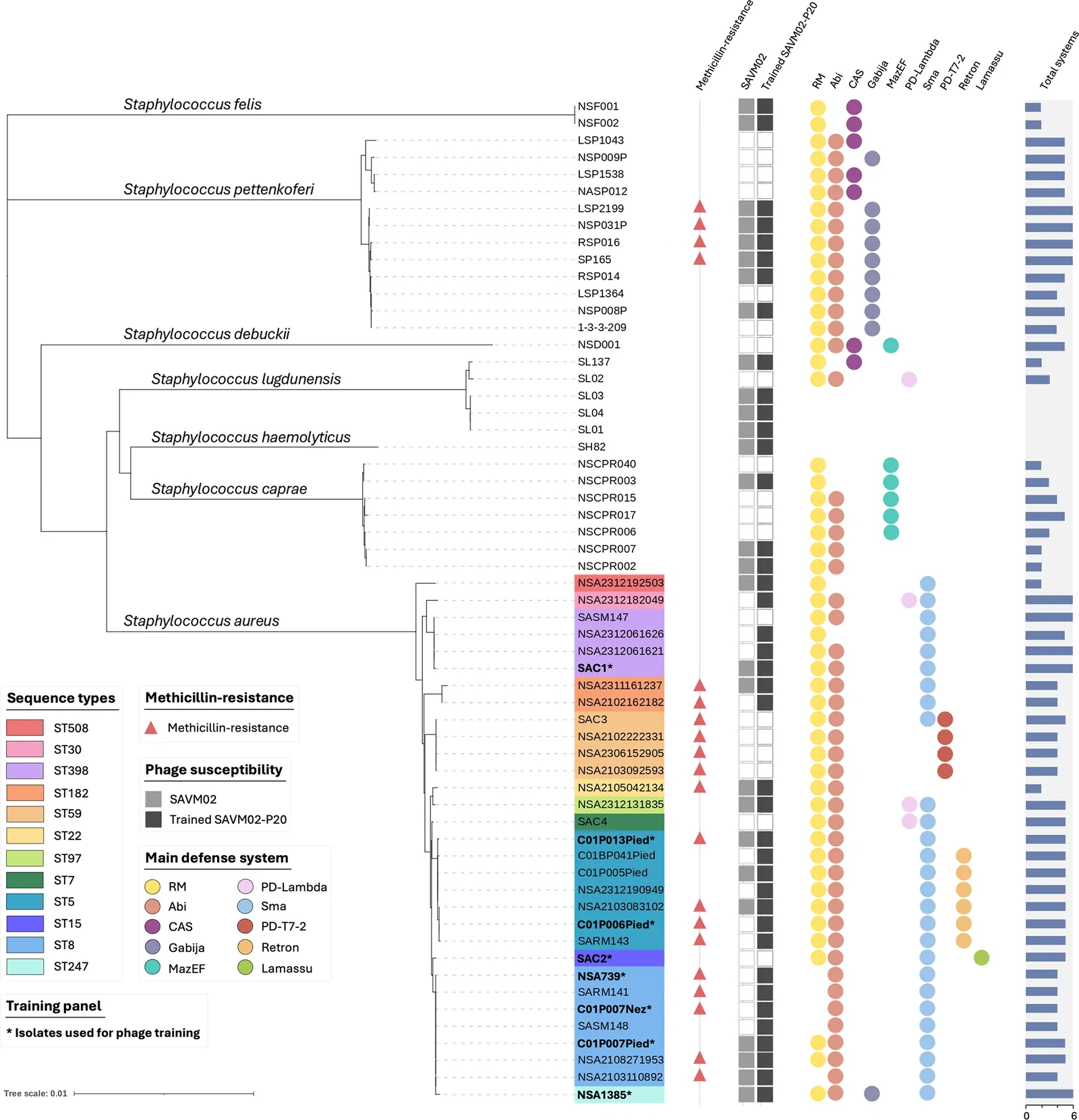
Whole-genome phylogenetic tree of clinical staphylococcal isolates showing their susceptibility to SAVM02 phages and the distribution of anti-phage defense systems. The tree includes 31 *S. aureus* and 28 CoNS. *S. aureus* sequence types (STs) are color-coded, and methicillin-resistant isolates are marked with red triangles. Susceptibility to the ancestral SAVM02 and trained SAVM02-P20 is indicated in light and dark gray squares, respectively. Isolates included in the phage training panel are indicated in bold with an asterisk and comprised four SAVM02-susceptible (NSA1385, SAC1, C01P007Pied, C01P013Pied) and four SAVM02 non-susceptible (NSA739, C01P007Nez, C1BP006Pied, SAC2) *S. aureus* isolates. The presence of selected anti-phage defense systems is shown as colored circles, including restriction-modification (RM), abortive infection (Abi), CRISPR-Cas (CAS), Gabija, MazEF, PD-Lambda, single-protein MazF-like antiphage (Sma), PD-T7-2, Retron, and Lamassu. The total number of defense systems detected per strain is indicated on the right.

WGS was performed on the 59 staphylococcal isolates to explore whether the infectivity profiles of the ancestral SAVM02 and trained SAVM02-P20 phages were associated with bacterial phylogenetic background. A neighbor-joining tree was constructed from the aligned sequences of all isolates, including 31 *S. aureus* strains and 28 CoNS. Within *S. aureus*, the isolates clustered into 12 STs, the most represented being ST398, ST59, ST5, and ST8 (Fig. 4). Ancestral SAVM02 exhibited activity against only a few isolates, scattered across different lineages (light gray). After training, SAVM02-P20 showed an expanded host range, with additional infected strains appearing across multiple STs (dark gray). Within both the ST5 and ST8 clusters, isolates included in the training protocol that were initially non-susceptible to SAVM02 became susceptible following training. In addition, SAVM02-P20 gained activity against several additional ST5 and ST8 isolates outside the training panel. Interestingly, none of the ST59 isolates (all MRSA, marked by the red triangles) were susceptible to either SAVM02 or SAVM02-P20, although this observation is based on limited strains. Among CoNS, susceptibility patterns remained largely unchanged following training and were not associated with any clear phylogenetic clustering.

The distribution of anti-phage defense systems was also examined across all isolates to assess their potential association with susceptibility patterns. In total, 20 defense systems were identified, of which 10 selected are shown in Fig. 4 and the remainder in Table S1. The number of systems per strain ranged from 0 to 6, with an average of 3.5 in CoNS and 4.6 in *S. aureus*. No clear relationship was observed between the number of defense systems and susceptibility to either SAVM02 or SAVM02-P20. Strains harboring multiple systems could still be infected (e.g., *S. aureus* SAC1 with six systems), while some strains with few systems remained resistant (e.g., *S. caprae* NSCPR040 with two systems), indicating that defense systems abundance alone does not fully explain resistance to phage infection. Only four strains in our data set were lacking detectable defense systems and correspondingly susceptible to phages. Common systems, such as restriction-modification (RM), predominantly type I, followed by type II and type IV, as well as abortive infection (Abi), were present in the majority of isolates, regardless of their susceptibility phenotype. Other systems, including CRISPR-Cas, Gabija, MazEF, single-protein MazF-like antiphage (Sma), PD-Lambda, and Retron, were more sporadically distributed and present in both phage susceptible and non-susceptible isolates. Nevertheless, some lineage or strain-specific patterns were observed. All ST59 isolates, which were non-susceptible to both phages, carried the PD-T7-2 system, while the *S. aureus* strain SAC2, also non-susceptible, was the only isolate harboring a Lamassu system. However, as these features are based on limited numbers, their contribution to phage resistance remains uncertain and should be further investigated.

### Phage training enhances replication efficiency on *S. aureus* isolates

**Fig 5:**
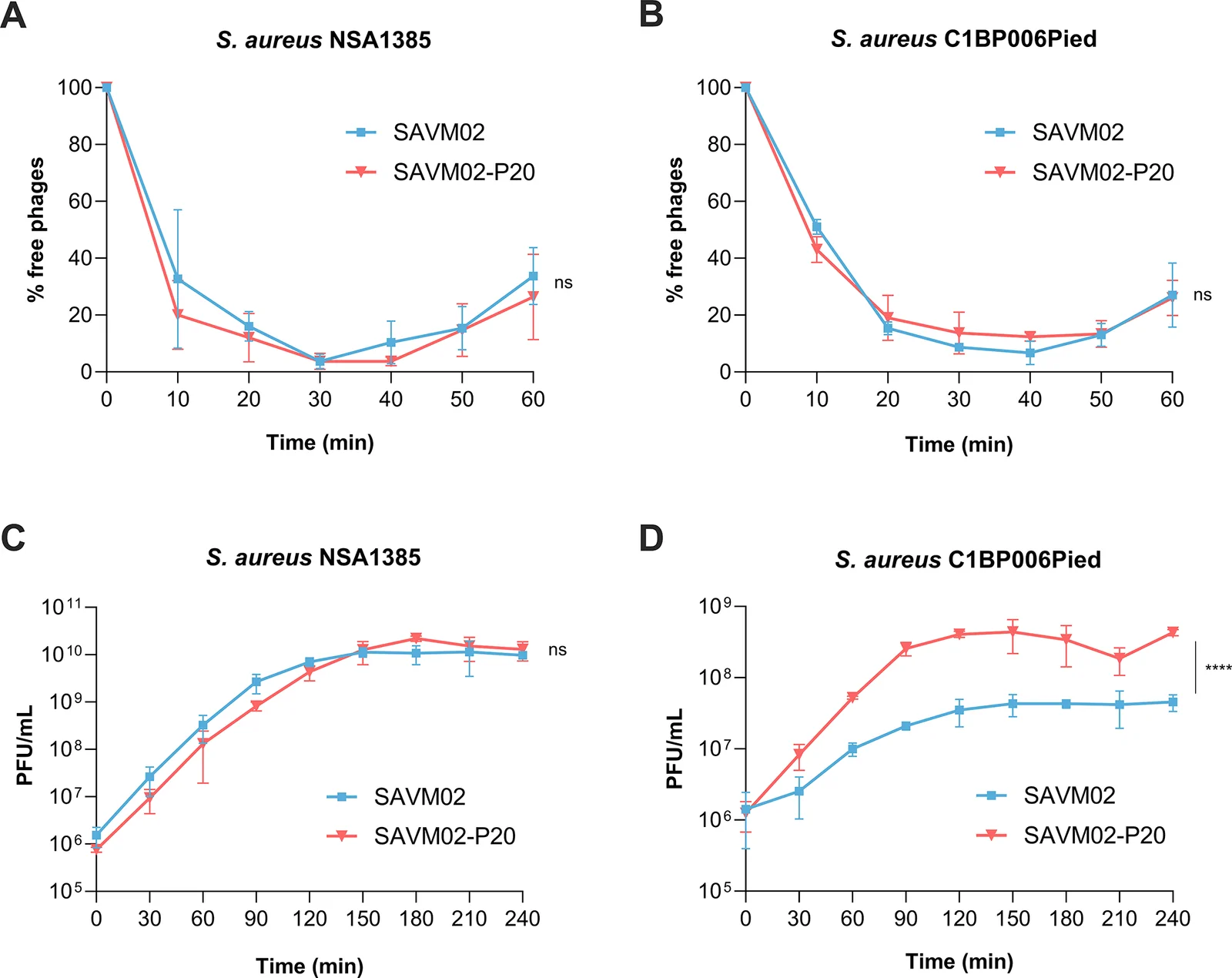
Adsorption and replication dynamics of ancestral SAVM02 and trained SAVM02-P20 phages on *S. aureus* isolates. Adsorption kinetics were monitored by quantifying the percentage of free phages over 60 min on NSA1385 (**A**) and C1BP006Pied (**B**). Replication dynamics were evaluated by measuring phage titers over 4 h on NSA1385 (**C**) and C1BP006Pied (**D**). Data represent mean values ± SD of three independent experiments. Statistical analyses were performed using two-way ANOVA (ns, not significant; ****P < 0.0001).

To determine whether the improved infectivity after phage training was linked to enhanced adsorption to host cells, we compared the proportion of free (non-adsorbed) phages over time for SAVM02 and SAVM02-P20 using two *S*. *aureus* strains, NSA1385 and C1BP006Pied, which are respectively susceptible and non-susceptible to SAVM02 as determined by EOP and LAS values. On NSA1385, both phages adsorbed rapidly, with more than 70% of particles bound within 10 min and less than 10% remaining free after 30 min (Fig. 5A). No substantial differences in adsorption kinetics were observed compared with the ancestral phage, consistent with the fact that NSA1385 is fully permissive. On the clinical isolate C1BP006Pied, adsorption occurred more slowly for both phages, with approximately 50% adsorbed after 10 min and 10–20% remaining free at 30 min (Fig. 5B). A slight increase in the proportion of free phages between 30 and 60 min likely reflects bacterial lysis and the release of newly produced virions. SAVM02-P20 did not show a significant increase in adsorption efficiency compared to SAVM02, suggesting that the gain in infectivity observed after training is likely attributable to downstream steps of the infection cycle rather than improved binding capacity.

In contrast, replication dynamics revealed a significant difference between the two phages. On NSA1385, SAVM02-P20, similar to SAVM02, reached ~10^10^ PFU/mL within 2–3 h (Fig. 5C). However, on C1BP006Pied, SAVM02-P20 achieved productive amplification, reaching 10^8^ and 10^9^ PFU/mL within 1–2 h, whereas SAVM02 reached only around 10^7^ PFU/mL after 4 h (Fig. 5D, ****P < 0.0001). These findings indicate that the improved infectivity of SAVM02-P20 is primarily driven by enhanced replication efficiency rather than by differences in adsorption.

### Evaluation of SAVM02-P20 in zebrafish embryos reveals strain-dependent therapeutic efficacy

To validate the therapeutic potential of the trained SAVM02-P20 phage *in vivo*, we employed a zebrafish embryo infection model with the two *S*. *aureus* strains, NSA1385 and C1BP006Pied. At 30 hpf, embryos were injected into the hindbrain ventricle with 10^4^ CFU/embryo of *S. aureus*. Two hours later, the same site was injected with phage suspensions at 10^9^ PFU/mL (MOI 0.1), corresponding to either the ancestral SAVM02, the trained SAVM02-P20, or heat-killed SAVM02-P20 (HK phage). Control embryos receiving PBS, SAVM02-P20 alone, or HK SAVM02-P20 alone showed a 100% survival rate, confirming the non-toxicity of the trained phage to zebrafish embryos and in line with previously reported results for SAVM02 (25).

**Fig 6:**
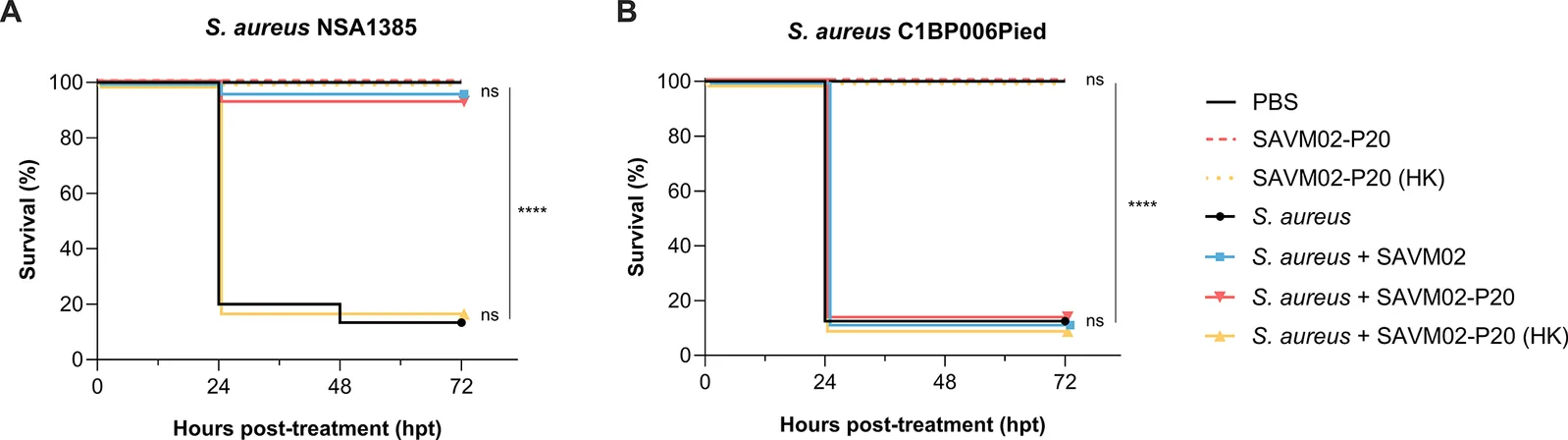
*In vivo* efficacy of SAVM02 and SAVM02-P20 in a zebrafish embryo infection model. Embryos at 30 hpf were injected in the hindbrain ventricle with 10^4^ CFU/embryo of *S. aureus* NSA1385 (**A**) or C1BP006Pied (**B**). Two hours later, embryos were treated in the same site with phage suspensions (10^9^ PFU/mL) of SAVM02, SAVM02-P20, or heat-killed (HK) phage. Control embryos receiving PBS, SAVM02-P20 alone, or HK SAVM02-P20 alone. Results are expressed as the proportion of surviving embryos (*n* = 30 per group, representative of three independent experiments). Survival curves were analyzed using the log-rank test (ns, not significant; ****P < 0.0001).

For NSA1385, untreated infection caused rapid mortality, with survival dropping to ~10% in 72 hpt (Fig. 6A). Both SAVM02 and SAVM02-P20 provided full protection, with survival rates comparable to the PBS controls, whereas HK phage failed to rescue embryos, confirming the requirement for active phage replication. For C1BP006Pied, infection was highly lethal, with survival decreasing to ~10% in 72 hpt in the untreated group. Neither SAVM02 nor SAVM02-P20 improved survival, which remained ~10% at 72 hpt (Fig. 6B). These results suggest that, under the tested conditions, SAVM02-P20 maintains efficacy against a fully susceptible strain *in vivo* but fails to improve survival against an initially non-susceptible strain.

### Phenotypic and genomic characterization of trained phage isolates reveals recurrent adaptive changes

**Fig 7:**
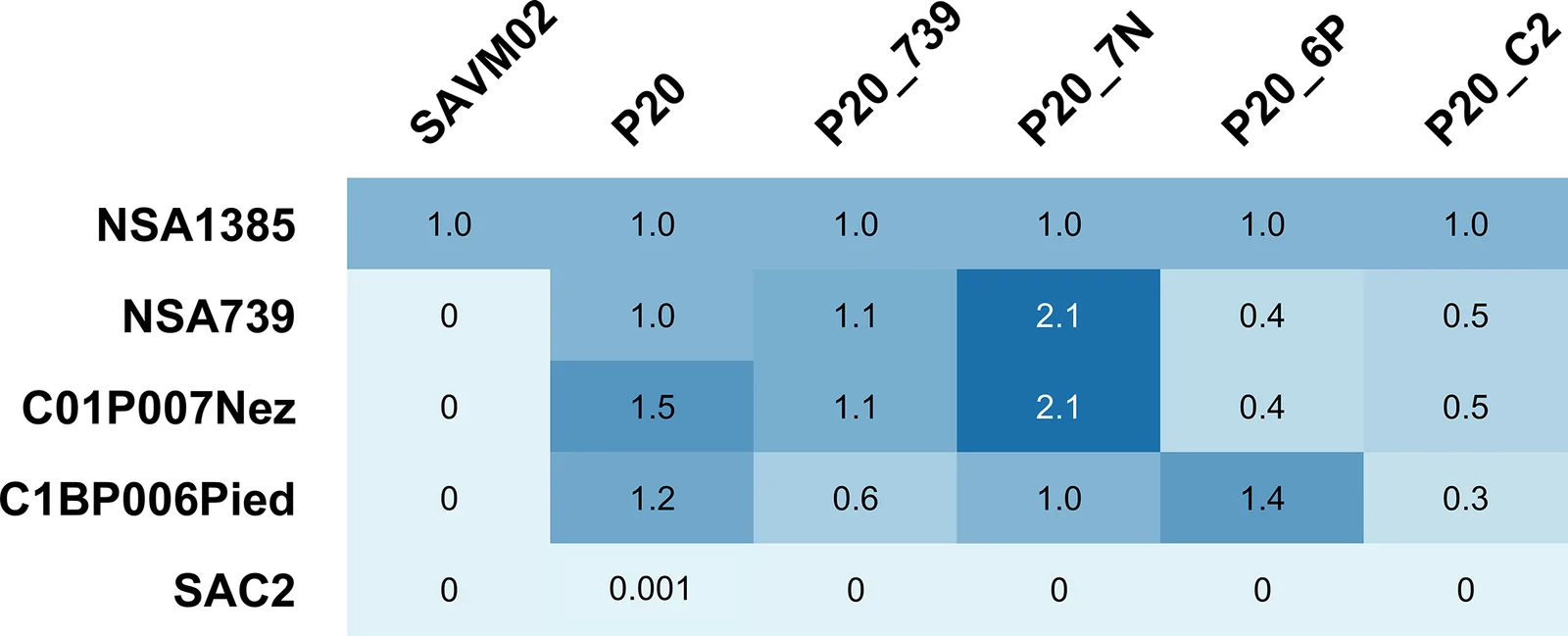
Activity of ancestral SAVM02, trained pool SAVM02-P20, and trained phage isolates against *S. aureus* isolates based on efficiency of plating (EOP). P20_739, P20_7N, P20_6P, and P20_C2 correspond to groups of plaque-purified phage isolates recovered from NSA739, C01P007Nez, C1BP006Pied, and SAC2, respectively. EOP values represent the mean of the three plaque-purified phage isolates recovered from each bacterial host. Strains were considered phage-susceptible when EOP was greater than 0.001.

To further explore the diversity and mechanisms underlying the improved infectivity of the trained phage, 12 plaque-purified phages isolates were recovered from the phage pool SAVM02-P20 by three successive rounds of plaque purification on each initially non-susceptible *S. aureus* strain included in the training protocol prior to amplification. Spot assays and EOP measurements performed against the SAVM02 propagation strain and the four initially non-susceptible *S. aureus* isolates revealed that all plaque-purified phage isolates retained infectivity against NSA1385, NSA739, C1P007Nez, and C1BP006Pied, while SAC2 remained fully resistant, as observed for SAVM02-P20 pool (Fig. 7). Although the trained phage isolates showed similar infectivity profiles, moderate variations in EOP were observed depending on the host strain, with some phage isolates exhibiting higher activity on specific bacterial isolates. In addition, spot assays performed on a subset of bacteria from the host range panel suggested that the overall infectivity profile of SAVM02-P20 was conserved among trained phage isolates.

To investigate the genomic mechanisms underlying the enhanced infectivity, WGS was performed on plaque-purified trained phage isolates for which high-quality genomic DNA was successfully obtained. Seven mutations were identified relative to the SAVM02 reference sequence (Table 1). These mutations included one stop-gain mutation, two frameshift mutations, one missense mutation, and one synonymous mutation affecting protein-coding sequences, as well as two non-coding mutations located in intergenic regions. Recurrent mutations were identified across all trained phage isolates. These included a frameshift insertion in a HU-like DNA-binding protein, as well as two non-coding mutations located upstream (−4 and −19 bp) of genes encoding an Ig-like protein and a phosphodiesterase. Although the HU-like mutation occurs at the very end of the coding sequence, affecting the last amino acid of the predicted 50-amino-acid protein, it was consistently detected across all trained phage isolates. Three additional mutations were detected among plaque-purified isolates recovered from three of the four bacterial hosts. Among them, a frameshift deletion affected a gene encoding a hypothetical protein, and a missense substitution resulted in an amino acid substitution near the N-terminus of a predicted AAA family ATPase. A synonymous substitution was also identified in a phage recombination exonuclease. Finally, a stop-gain mutation identified in another hypothetical protein was detected in only one plaque-purified isolate recovered from C01P007Nez. This mutation resulted in truncation of the predicted 97-amino acid protein after only six residues. Overall, several mutations recurred among plaque-purified phage isolates recovered from different bacterial hosts, suggesting that these genomic changes were maintained during phage training. While these genomic changes may contribute to the observed phenotype, their precise functional consequences cannot be inferred from sequence data alone.

**TABLE 1:** Mutations identified in plaque-purified phage isolates recovered from the SAVM02-P20 pool relative to the ancestral SAVM02 genome^*a*^

Position	Type	Nucleotide change	Functional consequence	Gene	P20 phage isolates recovered from								
					C1BP006Pied			C1BP007Nez			NSA739	SAC2	
					6P.1	6P.2	6P.3	7N.1	7N.2	7N.3	739.1	C2.1	C2.2
86039	SNP	G → T	Early stop-gain truncation	Hypothetical protein					X				
86167	Deletion	TC → T	Frameshift mutation	Hypothetical protein	X	X	X				X	X	X
86384	SNP	C → T	Synonymous substitution (Ala → Ala)	Phage recombination exonuclease	X	X	X				X	X	X
96569	Insertion	C → CT	Frameshift mutation near C-terminus	HU-like protein	X	X	X	X	X	X	X	X	X
104948	Complex	GA → AC	Non-coding upstream mutation	Ig-like protein	X	X	X	X	X	X	X	X	X
106526	Insertion	A → AACT	Non-coding upstream mutation	Phosphodiesterase	X	X	X	X	X	X	X	X	X
122510	SNP	G → T	Missense substitution near N-terminus (Gln → Lys)	AAA family ATPase	X			X	X		X		

^*a*^ Phage isolates are grouped according to the bacterial host from which they were recovered. “X” indicates the presence of the corresponding mutation; blank cells indicate its absence.

## DISCUSSION

Although the prevalence of multidrug-resistant *S. aureus* has declined in recent years, these strains still pose a major therapeutic challenge in DFIs, where conventional antibiotics often fail (26). Phage therapy has regained interest as a promising adjunctive approach, but its clinical use is frequently constrained by the narrow host range of phages. Among strategies to overcome this limitation, the Appelmans protocol described by Burrowes et al. (11) has emerged as a robust method to select phage variants with improved infectivity profiles through iterative co-culture with bacterial hosts. During this process, phages accumulate spontaneous genetic changes, such as point mutations or small insertions and deletions, that enhance their ability to infect otherwise resistant strains. In practice, this evolutionary training not only broadens the host range but may also delay or reduce the rise of phage-resistant bacteria during therapy (10). In this study, we adapted this approach for a single phage rather than multiple phages, performing directed evolution to broaden SAVM02 activity against clinical isolates from DFIs and DFOMs.

Our findings demonstrated that training progressively enhanced the activity of SAVM02 against previously non-susceptible DFIs/DFOMs isolates included in the training protocol. Both spot tests and liquid growth inhibition assays confirmed a gradual gain of activity over successive passages, consistent with previous reports that directed *in vitro* evolution can restore phage activity against resistant bacteria. For instance, Betts et al. (27) showed that phages evolved on *Pseudomonas aeruginosa* regained infectivity against resistant mutants, while Kunisch et al. (28) recently demonstrated that trained phages could more effectively target *P. aeruginosa* biofilms by exploiting resistance-associated weaknesses. Similar approaches have also been applied to gram-positive pathogens, with Lossouarn et al. (29) expanding the host range of phages infecting *Enterococcus faecium* and Kolenda et al. (30) adapting anti-*S*. *aureus* phages to infect *S. epidermidis*.

When expanding the analysis to a broader panel of clinical isolates, we observed that the phage pool SAVM02-P20 displayed a significantly expanded host range, infecting nearly 77% of *S. aureus* strains compared to 39% for SAVM02. This increased activity was distributed across multiple clonal complexes. Notably, gains in susceptibility were primarily observed within ST5 and ST8 clusters, including isolates outside the training panel. Because both permissive and non-permissive representatives of these STs were included during phage training, genetically related permissive hosts may have facilitated progressive adaptation toward otherwise non-susceptible strains by providing intermediate evolutionary backgrounds. By contrast, the distinct ST15 isolate SAC2, for which no permissive counterpart was included in the training panel, remained refractory throughout the protocol, as did isolates belonging to the ST59 lineage. These observations should nevertheless be interpreted with caution given the limited number of isolates tested, which may not fully capture the genetic diversity of the species. Despite this limitation, phage training broadened activity across diverse genetic backgrounds while remaining influenced by both strain-level and phylogenetic determinants of susceptibility.

To explore the basis of this variability, we examined the distribution of known anti-phage defense systems. No clear association was observed between susceptibility and either the number or identity of known defense systems, suggesting that resistance is unlikely to rely on a single mechanism. Nevertheless, specific systems were identified in some lineages isolates. The PD-T7-2 system identified in all ST59 isolates has been associated with resistance to multiple phages and is related to Sir2/HerA-like defense systems likely acting through disruption of cellular metabolism (e.g., NAD^+^ depletion) and inhibition of phage replication (31, 32). Similarly, the Lamassu system detected in the resistant isolate *S. aureus* SAC2 is thought to function via an abortive infection-like response, triggering infected cell death to block phage propagation (33). As these systems are restricted to a limited number of isolates, their contribution to phage resistance in our data set remains to be explored, suggesting that additional possibly uncharacterized factors may play a role. Together, these observations highlight the complexity of phage-host interactions and help explain the variability observed in training outcomes. This is consistent with previous reports showing that phage adaptation can improve infectivity while remaining constrained by strong intra- and inter-lineage differences (30). More broadly, phage training results are not always predictable, as adaptation may involve trade-offs between traits, such as replication efficiency and host range (10, 27).

To further investigate the mechanisms, adsorption and replication assays were performed to compare the ancestral and trained phage pools. Mechanistic assays suggested that improved infectivity of SAVM02-P20 was not associated with enhanced adsorption, as binding efficiency remained similar to that of the ancestral phage. Instead, replication assays revealed that the trained phage pool amplified more efficiently in poorly permissive hosts, pointing toward adaptive changes in downstream steps, such as genome replication, assembly, or evasion of bacterial defenses. Other studies have reached similar conclusions. Kolenda et al. (30) reported that phage adaptation from *S. aureus* to *S. epidermidis* was not associated with increased adsorption, and *Kayvirus* adaptation has likewise been linked to mutations in host takeover genes (13), suggesting that post-adsorption mechanisms may contribute to host range expansion in staphylococci. By contrast, adaptation of phages infecting gram-negative bacteria frequently involves receptor shifts. For example, Borin et al. (34) showed that trained *Escherichia coli* phages evolved to exploit alternative surface receptors when the original one was downregulated by the host. This strategy both broadened the host range and reduced the likelihood of resistance re-emergence by increasing the mutational burden required for escape. Taken together, these findings illustrate that phage training can follow distinct evolutionary trajectories depending on the bacterial species, converging nonetheless on enhanced infectivity and delayed resistance.

To complement these analyses, individual trained phages were isolated from the trained phage pool SAVM02-P20, phenotypically characterized, and sequenced to investigate the mechanisms underlying the improved infectivity of the trained phage isolates. Interestingly, trained phages isolated on distinct bacterial hosts retained the broadened infectivity profile of the SAVM02-P20 pool despite moderate variations in EOP depending on the tested strains. Rather than displaying strict host-specific specialization, trained phage isolates exhibited overall infectivity patterns, suggesting that the adaptive changes selected during phage training may confer broader benefits across multiple poorly permissive hosts. Similarly, laboratory adaptation of the therapeutic *S. aureus* phage Sb-1 on a resistant MRSA strain selected phage mutants with expanded host ranges (35).

In line with the adsorption and replication assays described above, several recurrent mutations affected genes involved in genome organization, DNA processing, or phage-host interactions, which may contribute to improved phage replication in previously non-permissive hosts. Interestingly, Lehman et al. (36) showed that loss-of-function mutations in gp102, a predicted DNA-binding and transcriptional regulatory protein of *S. aureus* phage K, improved antibacterial activity on poorly permissive USA300 strains without affecting adsorption. These findings further support the possibility that changes in post-adsorption processes may contribute to improved infectivity in staphylococcal phages. Among the mutations identified in our study, the frameshift mutation affecting the HU-like DNA-binding protein may, therefore, be of particular interest, as HU proteins are known to play a central role in DNA architecture and compaction (37). Although this mutation occurs at the very end of the coding sequence, its recurrence across all trained phage isolates suggests that even subtle alterations in this protein could influence phage genome organization and replication dynamics. Likewise, the missense mutation identified near the N-terminus of the AAA family ATPase may affect ATP-dependent processes involved in phage replication or genome packaging (38). The two non-coding mutations located upstream of the Ig-like protein (−4 bp) and phosphodiesterase gene (−19 bp) may also be relevant, as they occur near translation and promoter regions, respectively, and could potentially affect gene expression. This may influence functions associated with host interaction in the case of the Ig-like protein, which is commonly associated with phage structural components and interactions with bacterial surface carbohydrates (39). Similarly, altered expression of the phosphodiesterase could affect anti-defense mechanisms, as phage-encoded phosphodiesterases have recently been shown to act as anti-defense proteins by degrading cyclic nucleotide signals involved in bacterial phage defense systems, such as CBASS, thereby preventing activation of antiviral responses and promoting phage replication (40). The recurrence of several mutations across trained phage isolates supports their potential contribution to the observed phenotype, although their individual effects remain unclear. It is nevertheless possible that some mutations represent neutral hitchhiking events associated with a smaller number of functionally relevant adaptive changes.

While the *in vitro* results demonstrated the benefits of phage training, validation in physiologically relevant models remains essential. Using the zebrafish embryo model, which has been developed for studying *S. aureus* phage interactions (24, 41), we observed that SAVM02-P20 showed no toxicity and retained full efficacy against a highly permissive strain but failed to improve survival in embryos infected with an initially poorly permissive isolate. This discrepancy illustrates that gains observed *in vitro* do not necessarily translate into therapeutic benefit *in vivo*, where additional factors, such as bacterial virulence, host immune responses, and phage pharmacokinetics, likely play a decisive role. Only very few studies have so far combined phage training through directed evolution with subsequent testing in animal models. Most published works have evaluated trained phages exclusively *in vitro* (27–30), whereas *in vivo* phage therapy studies have generally relied on non-trained phages or cocktails (7, 41). A notable exception is the preclinical work of Morello et al. (12) who demonstrated that a phage optimized through five consecutive passages on a single clinical *P. aeruginosa* strain exhibited markedly improved efficacy both *in vitro* and in a murine infection model. However, their approach differed from ours, as the training procedure was performed on a single bacterial host background, and the resulting adapted phage was subsequently isolated and characterized individually. By contrast, our study aimed to expand host range across multiple *S. aureus* clinical isolates using a multi-host training strategy and primarily evaluated the trained phage pool SAVM02-P20. Differences in training design and phage population composition may, therefore, contribute to differences in therapeutic performance. Our findings nevertheless not only contribute to this emerging evidence base, but also highlight that the benefits of training may vary substantially depending on the host-phage-environment interplay. Further optimization, through dosing, timing, or phage combinations, will likely be required to translate evolutionary gains into consistent therapeutic success.

Finally, this study has several limitations. Although our panel included both *S. aureus* and CoNS isolates collected over several years, it remains insufficient to reflect the full genetic diversity of DFI/DFOM isolates. For instance, ST59 remained refractory to SAVM02-P20, illustrating the influence of lineage-specific factors. A more representative panel would require broader geographic sampling, larger number of isolates per species, and inclusion of other clinically relevant staphylococci, such as *S. epidermidis*, which also plays a significant role in DFIs (42). Another limitation is the lack of evaluation of long-term stability of the trained phage activity. Although infectivity was maintained after limited amplification on permissive hosts, suggesting short-term stability, the persistence of this phenotype over extended passages and the potential reversion in the absence of selective pressure were not assessed. Future studies should also determine whether the stabilization of individual trained phage clones, rather than the maintenance of heterogeneous trained pools, could improve the long-term preservation of adaptive phenotypes. As pointed out by Oromí-Bosch et al. (10), restoring infectivity is only part of the challenge, while preventing rapid resistance re-emergence is equally critical. More broadly, these authors argue that phage therapy strategies should not only maximize antibacterial activity but also steer bacterial evolution toward beneficial trade-offs, such as reduced virulence or renewed antibiotic susceptibility. Although these aspects were beyond the scope of the present study, they represent important directions for future work. For example, combining trained phages with others targeting essential virulence determinants could force *S. aureus* into evolutionary dead-ends that favor therapeutic success. Future optimization of phage training strategies may also benefit from the simultaneous evolution of multiple complementary phages, as well as a more rational selection of bacterial hosts included in the training protocol.

Altogether, our findings demonstrate that phage training can substantially expand the host range of a therapeutic phage while defining the boundaries of this approach. Rather than a universal solution, training should be considered as one component of a broader therapeutic strategy. In practice, trained phages could be generated in advance against local collections of isolates to build phage libraries or produced on demand for patient-specific strains in a personalized approach (43). A clinical proof-of-concept was provided by Ujmajuridze et al. (44), who successfully updated a phage cocktail targeting multiple uropathogens through training, increasing coverage of clinical isolates from 41 to 75% and achieving bacterial load reduction in most treated patients. Such findings illustrate that leveraging training to expand host range can not only simplify cocktail formulation but also accelerate translation into clinical use. In addition, the localized administration of phage therapy in DFIs may help mitigate potential off-target effects associated with expanded host range (26). Importantly, trained phages arise through spontaneous mutations rather than genetic engineering, which may facilitate their regulatory acceptance as natural variants (43, 45). Altogether, our findings support phage training as a promising but complementary tool to overcome bacterial resistance in complex infections, such as DFIs/DFOMs. Future work should focus on optimizing this approach through larger panels, stability studies, and integration with strategies that exploit evolutionary trade-offs, ultimately paving the way for robust, personalized phage therapy.

## Data Availability

WGS data of staphylococcal isolates have been deposited in the NCBI database under BioProject accession number PRJNA1456407. Raw sequencing reads of trained phage isolates and the SAVM02 wild-type phage have been deposited in the European Nucleotide Archive under BioProject accession number PRJEB106256.

## SUPPLEMENTAL MATERIAL

Supplemental material aac.00639-26-s0001.pdf Fig. S1; Table S1.

ASM does not own the copyrights to Supplemental Material that may be linked to, or accessed through, an article. The authors have granted ASM a non-exclusive, world-wide license to publish the Supplemental Material files. Please contact the corresponding author directly for reuse.
