# Supplementary material for: Training a phage to expand its host range: directed evolution against Staphylococcus aureus from diabetic foot infections
Source: Antimicrob Agents Chemother. 2026 Aug 24;70(10):e00639-26. doi: 10.1128/aac.00639-26 (PMC13689060; doi:10.1128/aac.00639-26)
Supplement: Supplementary file 1 [file aac.00639-26-s0001.pdf]

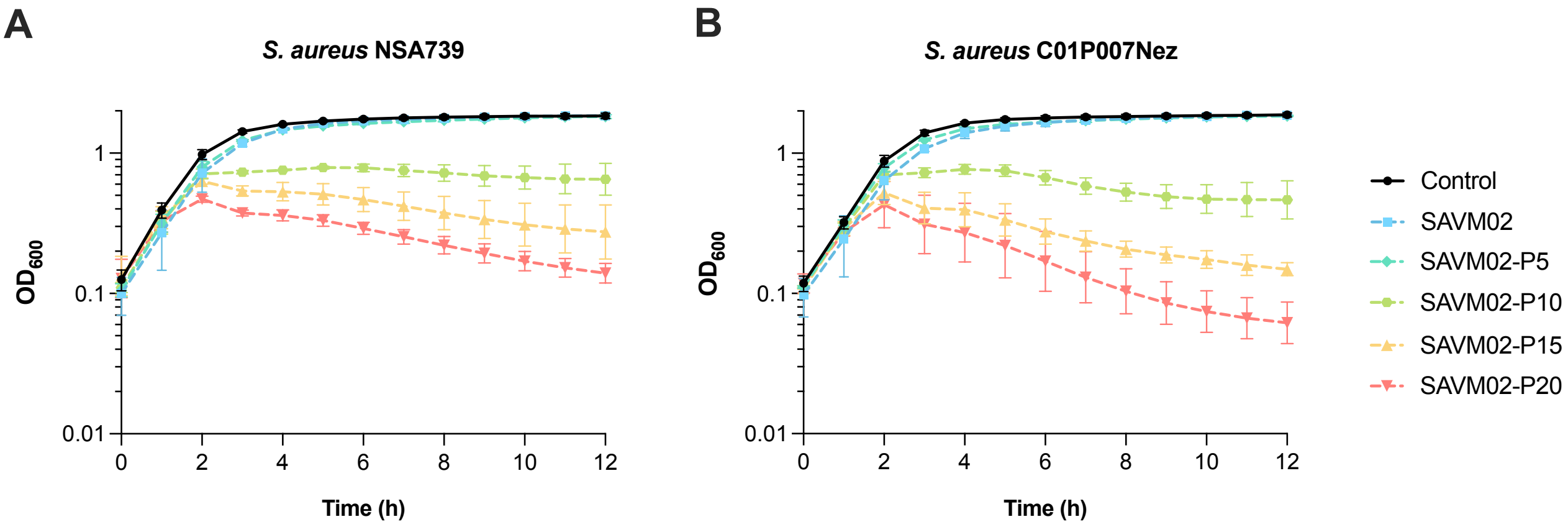

Figure S1. Bacterial growth inhibition by ancestral SAVM02 phage and trained phage pools on *S. aureus* isolates. Growth curves are shown for NSA739 **(A)** and C01P007Nez **(B)**, which both exhibited progressive susceptibility to trained phages, similar to the profile observed for C1BP006Pied in the main manuscript (Fig. 2B). Bacterial growth was monitored over 12 h in the presence of the ancestral SAVM02 phage, trained phage pools (P5, P10, P15, P20), or without phage (control). Data represent mean OD_{600} readings ± SD from three independent experiments. Corresponding LAS values for these isolates are summarized together with the full panel in the main manuscript (Fig. 2D).

Table S1. Distribution of phage susceptibility and predicted anti-phage defense systems among clinical staphylococcal isolates. Clinical staphylococcal isolates are listed along with their associated metadata (species, isolate name, sequence type for *S. aureus*, collection date, and location). Phage susceptibility to SAVM02 and SAVM02-P20, as well as the presence of predicted anti-phage defense systems in isolates, are indicated by "X". Abbreviations: RM, restriction-modification; Abi, abortive infection; CAS, CRISPR-Cas ; Sma, single-protein MazF-like antiphage.

Clinical isolates		Sequence types	Collection date	City	Phage activity		Phage defense systems																			
					SAVM02	SAVM02-P20	RM	Abi	CAS	Gabija	RosmerTA	SoFIC	Bunzi	RloC	Theoris	MazEF	PD-Lambda	Gao-Her	Cernunnos	CBASS	Sma	PD-T7-2	Retron	Lamassu	Dodola	Avs
S. felis	NSF001		2024	Nîmes, FR	X	X	X		X																	
	NSF002		2024	Nîmes, FR	X	X	X		X																	
S. pettenkoferi	LSP1043		2019	Armentières, FR			X	X	X		X	X														
	NSP009P		2020	Nîmes, FR			X	X		X	X		X													
	LSP1538		2018	Tourcoing, FR			X	X	X		X			X												
	NASP012		2024	Nantes, FR			X	X	X		X			X												
	LSP2199		2020	Lyon, FR	X	X	X	X		X	X	X	X													
	NSP031P		2022	Nîmes, FR	X	X	X	X		X	X	X	X													
	RSP016		2022	Rennes, FR	X	X	X	X		X	X	X	X													
	SP165		2018	Nîmes, FR	X	X	X	X		X	X	X	X													
	RSP014		2022	Rennes, FR	X	X	X	X		X	X	X														
	LSP1364		2022	Rodez, FR			X	X		X	X															
	NSP008P		2020	Nîmes, FR	X	X	X	X		X	X	X														
	1-3-3-209		2019	Geneva, CH			X	X		X	X															
S. debuckii	NSD001		2022	Nîmes, FR			X	X	X						X	X										
S. lugdunensis	SL137		2018	Nîmes, FR	X	X	X		X																	
	SL02		2022	Nîmes, FR			X	X									X									
	SL03		2022	Nîmes, FR	X	X																				
	SL04		2022	Nîmes, FR	X	X																				
	SL01		2022	Nîmes, FR	X	X																				
	SH82		2018	Nîmes, FR	X	X																				
S. caprae	NSCPR040		2020	Nîmes, FR			X									X										
	NSCPR003		2020	Nîmes, FR	X	X	X									X		X								
	NSCPR015		2021	Nîmes, FR			X	X								X		X								
	NSCPR017		2022	Nîmes, FR			X	X								X			X	X						
	NSCPR006		2021	Nîmes, FR			X	X								X										
	NSCPR007		2021	Nîmes, FR	X	X	X	X																		
	NSCPR002		2021	Nîmes, FR	X	X	X	X																		
S. aureus	NSA2312192503	ST508	2023	Nîmes, FR	X	X	X														X					
	NSA2312182049	ST30	2023	Nîmes, FR		X	X	X			X			X			X				X					
	SASM147	ST398	2018	Nîmes, FR			X	X			X			X	X						X					
	NSA2312061626		2023	Nîmes, FR		X	X				X			X	X						X					
	NSA2312061621		2023	Nîmes, FR		X	X	X			X			X	X						X					
	SAC1		2021	Nîmes, FR	X	X	X	X			X			X	X						X					
	NSA2311161237	ST182	2023	Nîmes, FR	X	X	X	X			X										X					
	NSA2102162182		2021	Nîmes, FR		X	X	X			X										X					
	SAC3	ST59	2021	Nîmes, FR			X	X			X										X	X				
	NSA2102222331		2021	Nîmes, FR			X	X			X											X				
	NSA2306152905		2023	Nimes, FR			X	X			X											X				
	NSA2103092593		2021	Nîmes, FR			X	X			X											X				
	NSA2105042134	ST22	2021	Nîmes, FR	X	X	X	X																		
	NSA2312131835	ST97	2023	Nîmes, FR	X	X	X	X			X						X				X					

	SAC4	ST7	2021	Nîmes, FR			X	X			X						X				X					
	C01P013Pied		2010	Nîmes, FR	X	X	X	X			X									X	X					
	C01BP041Pied		2011	Nîmes, FR		X	X	X			X										X		X			
	C01P005Pied		2010	Nîmes, FR	X	X	X	X			X										X		X			
	NSA2312190949	ST5	2023	Nîmes, FR		X	X	X			X										X		X			
	NSA2103083102		2021	Nîmes, FR	X	X	X	X			X										X		X			
	C1BP006Pied		2010	Nîmes, FR		X	X	X			X										X		X			
	SARM143		2018	Nîmes, FR		X	X	X			X										X		X			
	SAC2	ST15	2021	Nîmes, FR			X	X			X										X			X		
	NSA739		2006	Nîmes, FR		X		X													X				X	X
	SARM141		2015	Nîmes, FR		X		X													X				X	X
	C01P007Nez		2010	Nîmes, FR		X		X													X				X	X
	SASM148	ST8	2018	Nîmes, FR		X		X													X				X	X
	C01P007Pied		2010	Nîmes, FR	X	X	X	X													X				X	X
	NSA2108271953		2021	Nîmes, FR	X	X	X	X													X				X	X
	NSA2103110892		2021	Nîmes, FR	X	X		X													X				X	X
	NSA1385	ST247	2006	Nîmes, FR	X	X	X	X		X											X				X	X
